# Supplementary figures and images for: Live-cell RNA imaging with the inactivated endonuclease Csy4 enables new insights into plant virus transport through plasmodesmata
Source: PLoS Pathog. 2025 Apr 9;21(4):e1013049. doi: 10.1371/journal.ppat.1013049 (PMC12052393; doi:10.1371/journal.ppat.1013049)

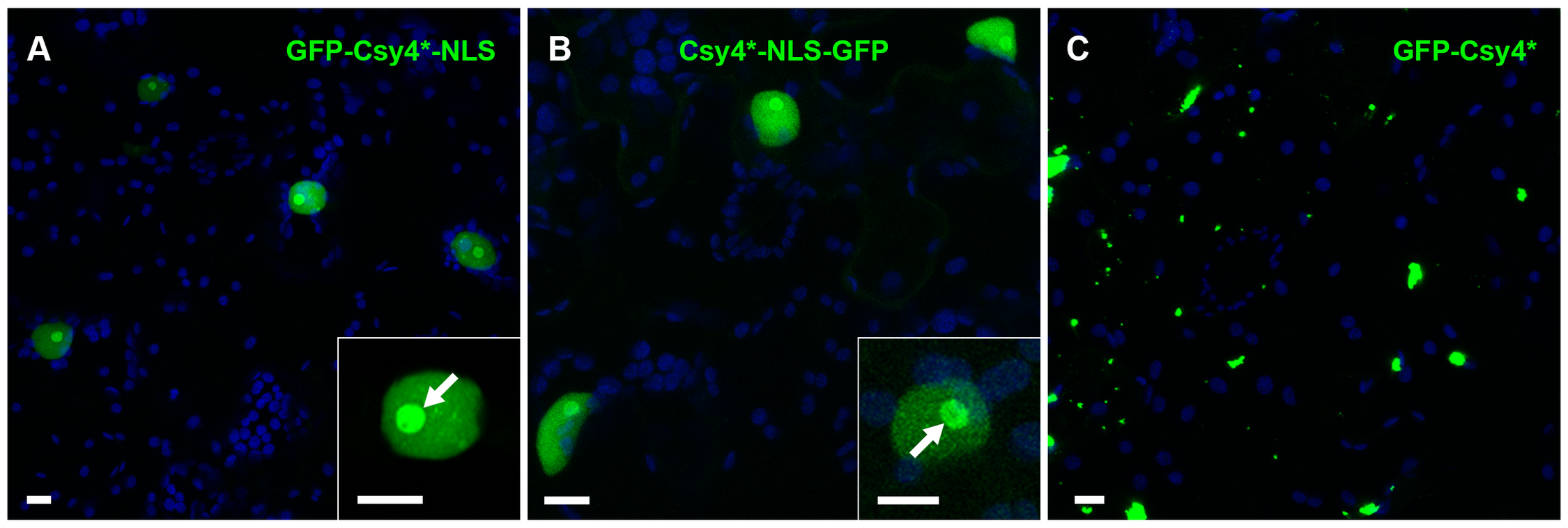

Supplement: S1 Fig — (A) N-terminal GFP fusion with nuclear localisation signal (NLS) shows exclusively nuclear localisation. Inset shows enrichment in nucleolus (arrow). (B) C-terminal GFP fusion with NLS shows exclusively nuclear localisation. Inset shows enrichment in nucleolus (arrow). (C) N-terminal GFP fusion without a NLS shows protein aggregates. All images are maximum intensity z-projections. GFP fluorescence shown green, chlorophyll auto-fluorescence shown blue. Scale bars, 10 µm. (TIF) [file ppat.1013049.s001.tif]

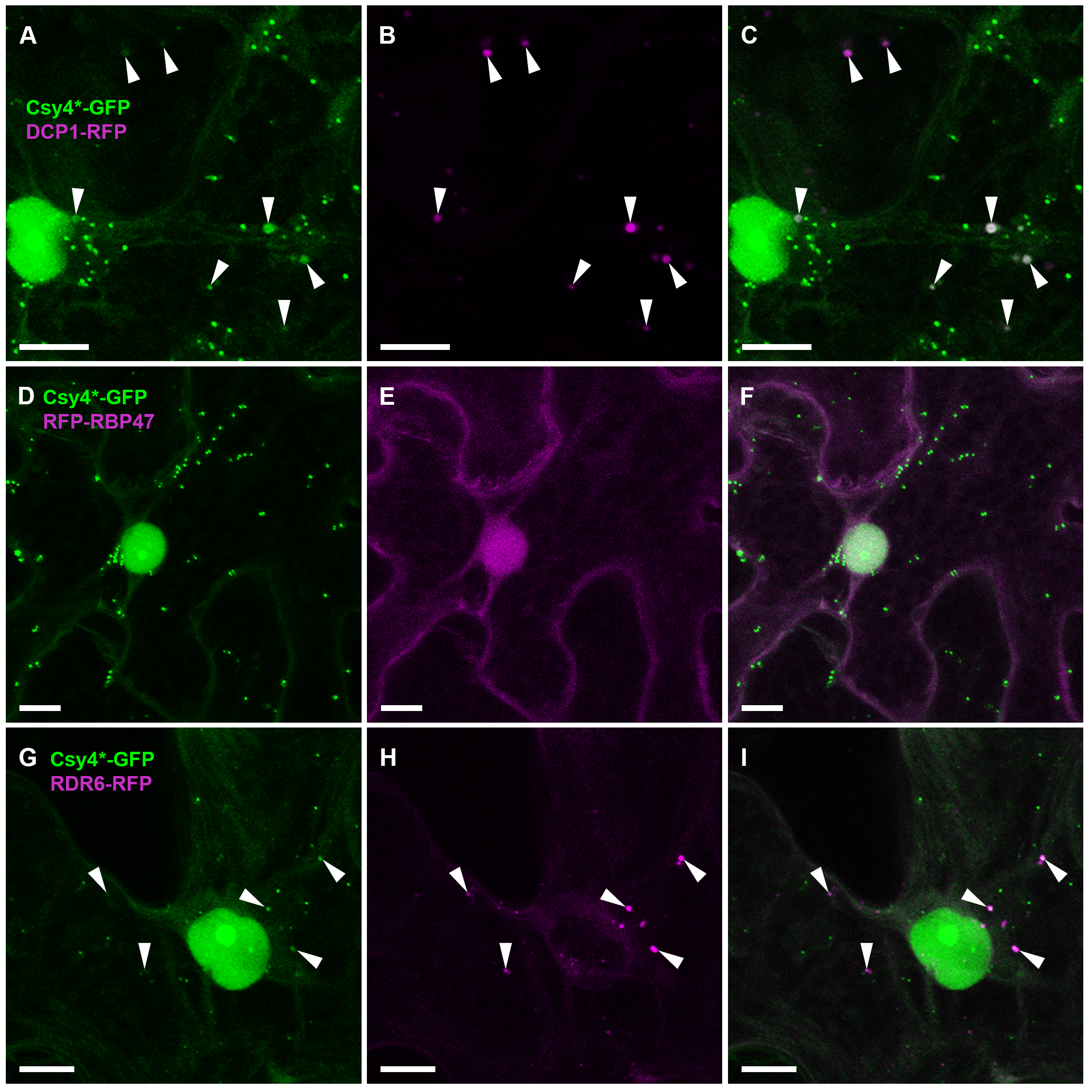

Supplement: S2 Fig — (A-C) Co-localisation with P-body marker DCP1. Arrow heads indicate DCP1 granules co-localised with Csy4*-GFP. (D-F) Co-localisation with stress granule marker RBP47. (G-I) Co-localisation with tasiRNA processing body marker RDR6. Arrow heads indicate RDR6 granules co-localised with Csy4*-GFP. Left column: GFP channel, middle column: RFP channel, right column: merge. GFP fluorescence shown green, RFP fluorescence shown magenta. All images are maximum intensity z-projections. Scale bars, 10 µm. (TIF) [file ppat.1013049.s002.tif]

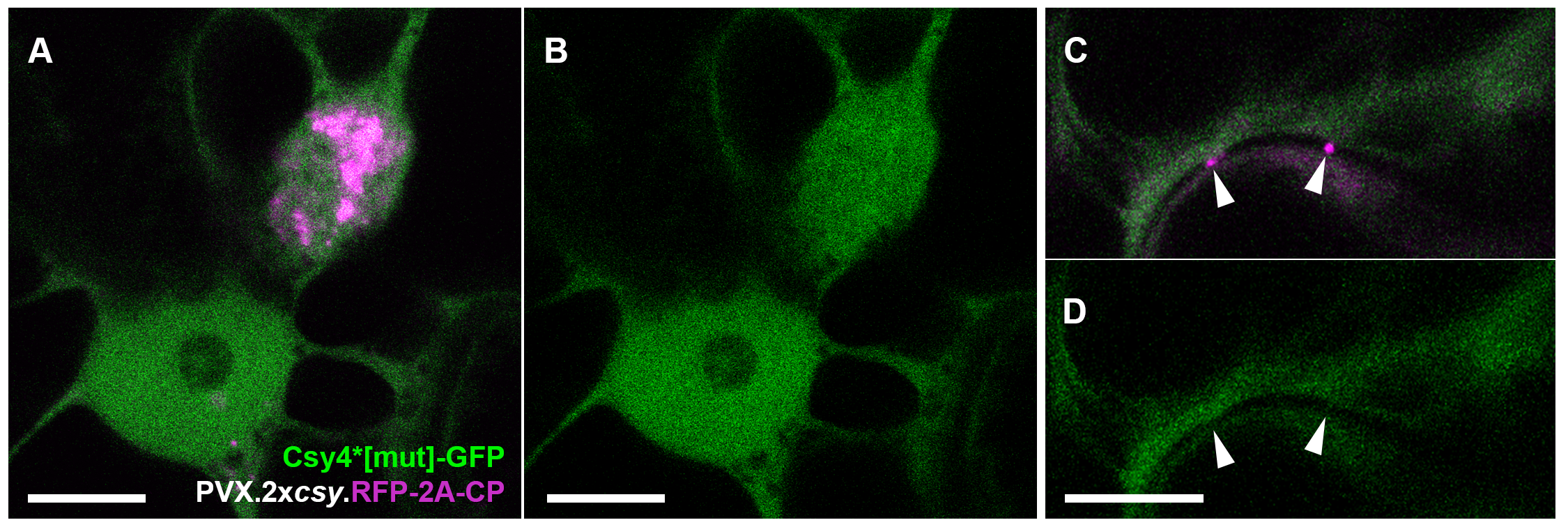

Supplement: S3 Fig — (A-B) Perinuclear VRC. GFP fluorescence is diffuse with no ‘whorls’ and Csy4*[mut]-GFP is not enriched in the nucleolus. (C-D). Absence of GFP fluorescence from RFP-2A-CP labelled PD (arrow heads). GFP fluorescence shown green, RFP fluorescence shown magenta. Image in (A,B) is a single z-section; image in (C,D) is a maximum intensity projection of three z-sections. Scale bars, 10 µm. (TIF) [file ppat.1013049.s003.tif]

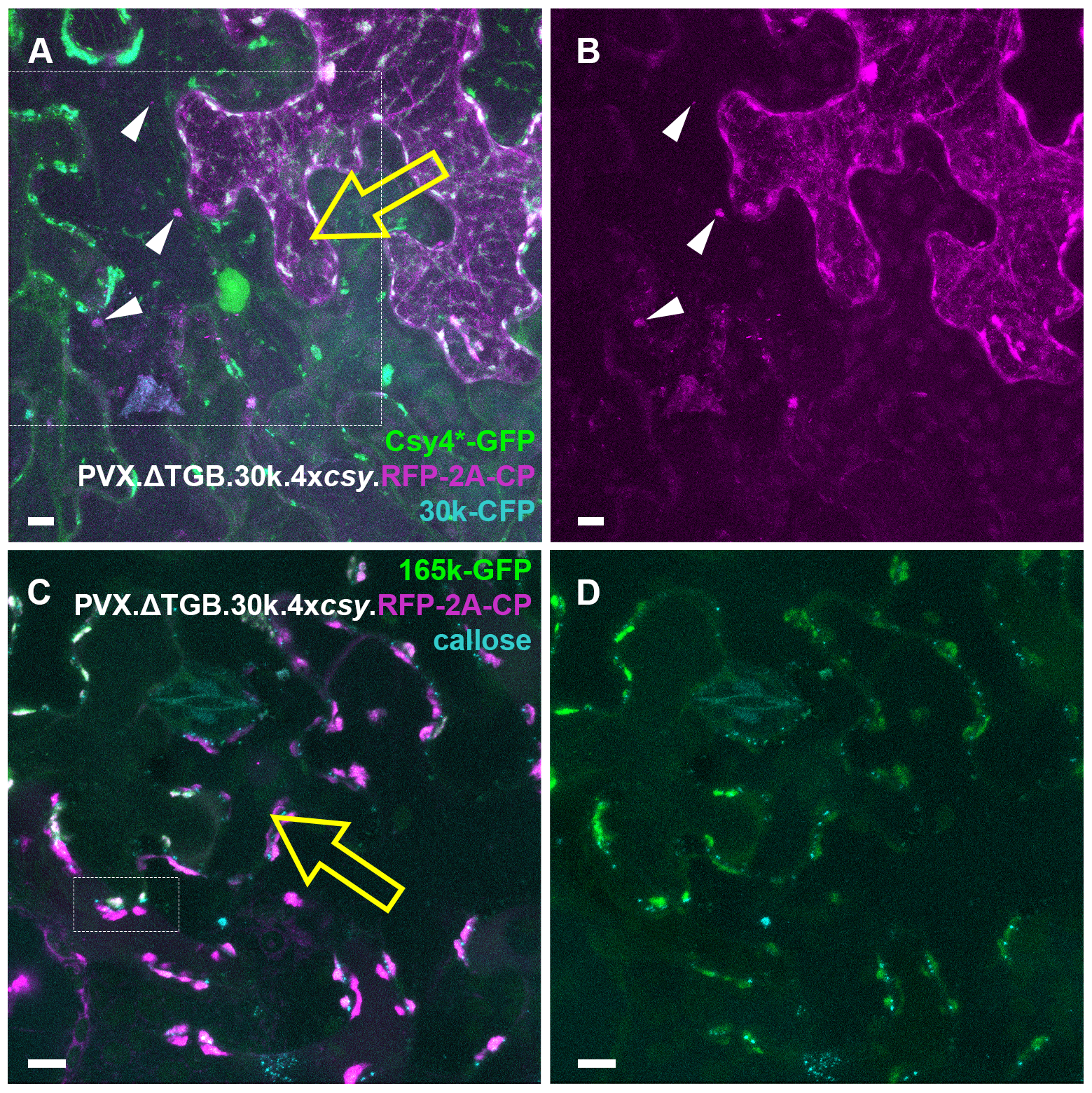

Supplement: S4 Fig — (A-B) Full maximum intensity projection of the entire z-stack used in Fig 6E (which is a projection of two z-sections). RFP fluorescence indicating PVX infection is visible in the second cell from the left (arrow heads). (C-D) Full image used for Fig 6F shows a multitude of PD-associated structures containing both RFP-2A-CP and 165k-GFP. Arrows: approximate direction of viral spread. Dashed boxes: regions enlarged in Fig 6E, F, respectively. GFP fluorescence shown green, RFP fluorescence shown magenta, CFP and aniline blue fluorescence shown cyan. All images are maximum intensity z-projections. Scale bars, 10 µm. (TIF) [file ppat.1013049.s004.tif]

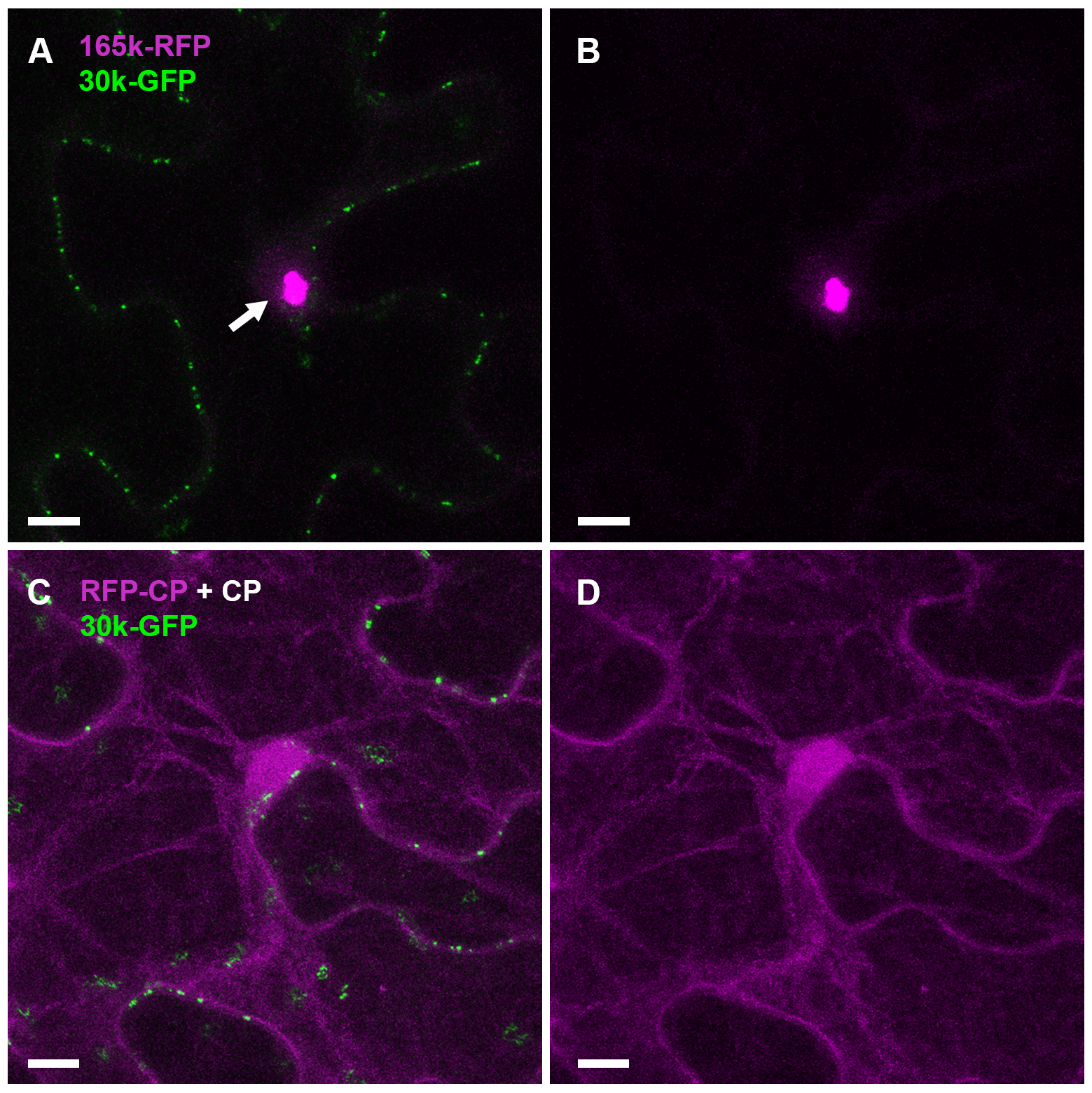

Supplement: S5 Fig — (A-B) Transient co-expression by agroinfiltration of 165k-RFP and 30k-GFP in N. benthamiana leaf epidermis. 165k-RFP remains confined to the nucleus with enrichment in the nucleolus (arrow). (C-D) Transient co-expression by agroinfiltration of RFP-CP, unfused CP and 30k-GFP in N. benthamiana leaf epidermis. RFP-CP remains nucleo-cytoplasmically distributed with no enrichment at PD or PD-adjacent structures. GFP fluorescence shown green, RFP fluorescence shown magenta. All images are maximum intensity z-projections. Scale bars, 10 µm. (TIF) [file ppat.1013049.s005.tif]

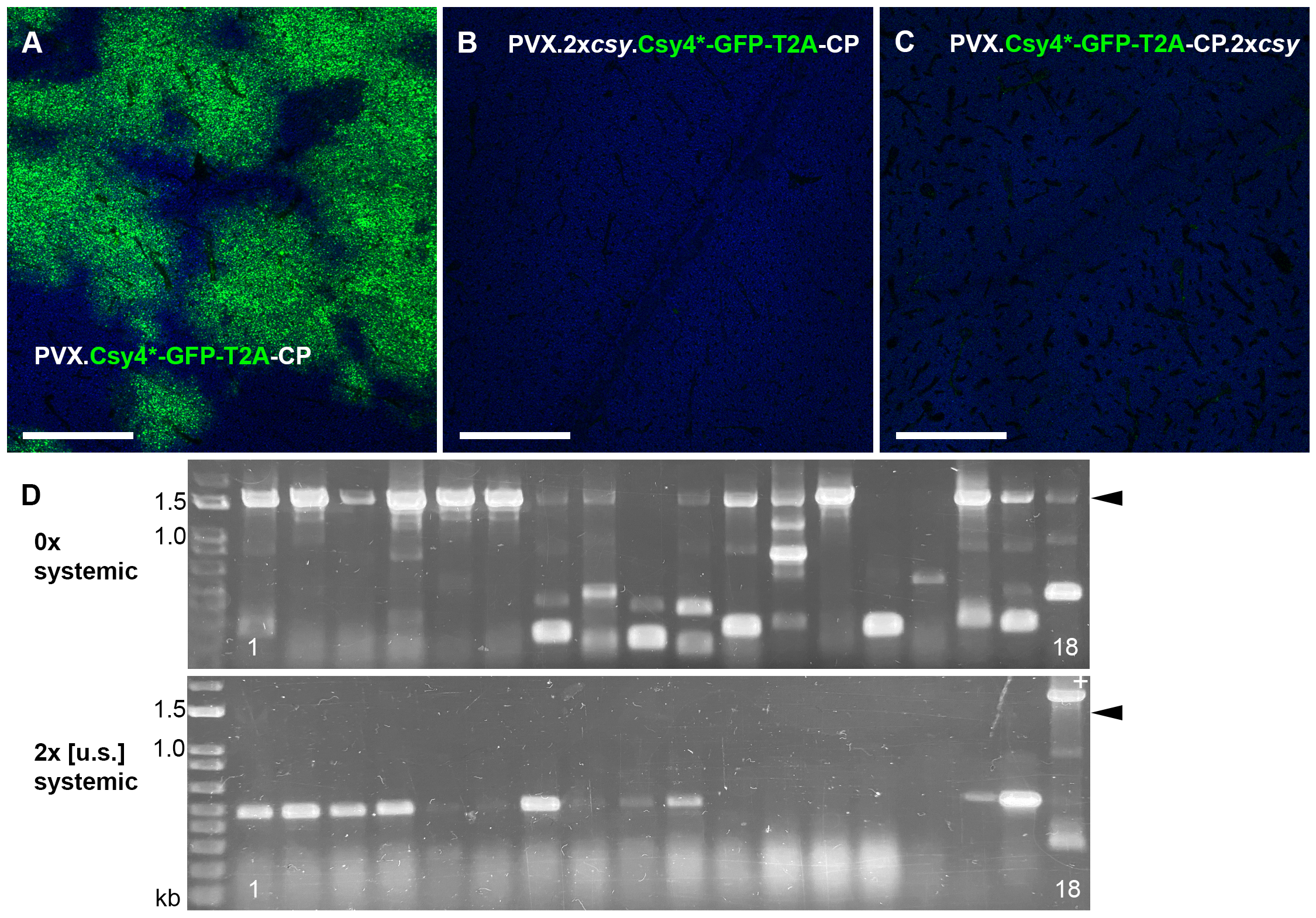

Supplement: S6 Fig — (A-C) Representative images from systemic leaves of plants infected with untagged PVX.Csy4*-GFP-T2A-CP (A), upstream-tagged PVX.2xcsy.Csy4*-GFP-T2A-CP (B) and downstream-tagged PVX.Csy4*-GFP-T2A-CP.2xcsy (C), respectively, at 14 days post inoculation (dpi). GFP fluorescence shown green, chlorophyll autofluorescence shown blue. All images are maximum intensity z-projections. Scale bars, 1 mm. (D) RT-PCR analysis of systemically infected leaves at 14 dpi. A segment of the PVX genome from the end of the TGB3 ORF to the beginning of the CP ORF was amplified. Arrow heads on right indicate expected product size when complete Csy4*-GFP-T2A-CP ORF is present. 1 to 18: three biological replicates with six plants each. 0x: untagged PVX.Csy4*-GFP-T2A-CP, 2x [u.s.]: upstream-tagged PVX.2xcsy.Csy4*-GFP-T2A-CP. (TIF) [file ppat.1013049.s006.tif]

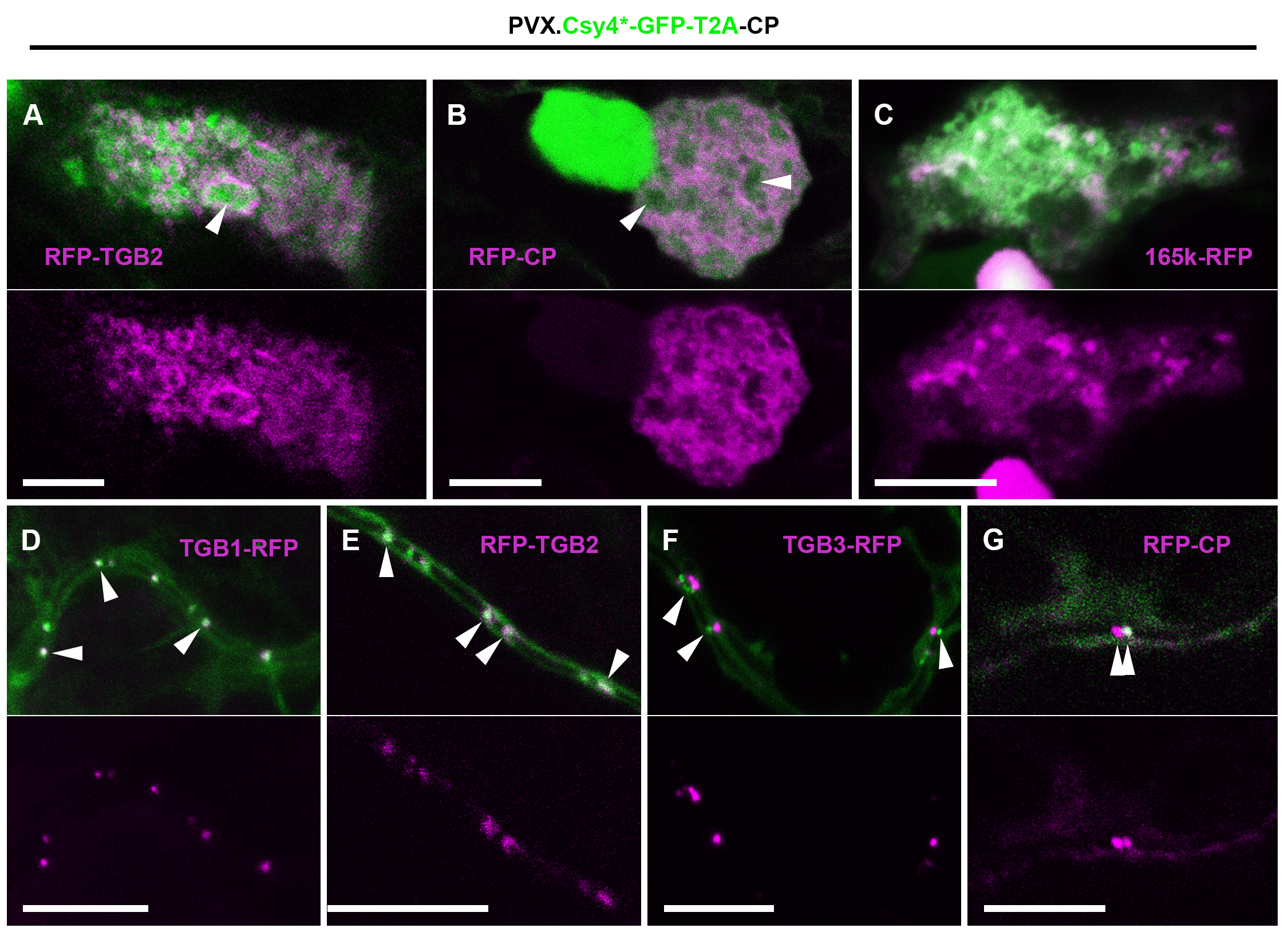

Supplement: S7 Fig — (A) RFP-TGB2 localises to the VRC, appearing to surround vRNA (arrow head). (B) RFP-CP localises to the VRC, also surrounding vRNA (arrow heads). (C) 165k-RFP replicase marker localises to granular structures among the vRNA in the VRC. (D, E and G) TGB1-RFP (D), RFP-TGB2 (E) and RFP-CP (G) co-localise with vRNA inside plasmodesmata (arrow heads). (F) TGB3-RFP is located in membrane structures at plasmodesmata entrances, separate from vRNA inside the channels (arrow heads). Top rows, merged images, bottom rows, RFP channel only. GFP fluorescence shown green, RFP fluorescence shown magenta. All images are single z-sections, except (F) which is a maximum intensity projection of two z-sections. Scale bars, 10 µm. (TIF) [file ppat.1013049.s007.tif]

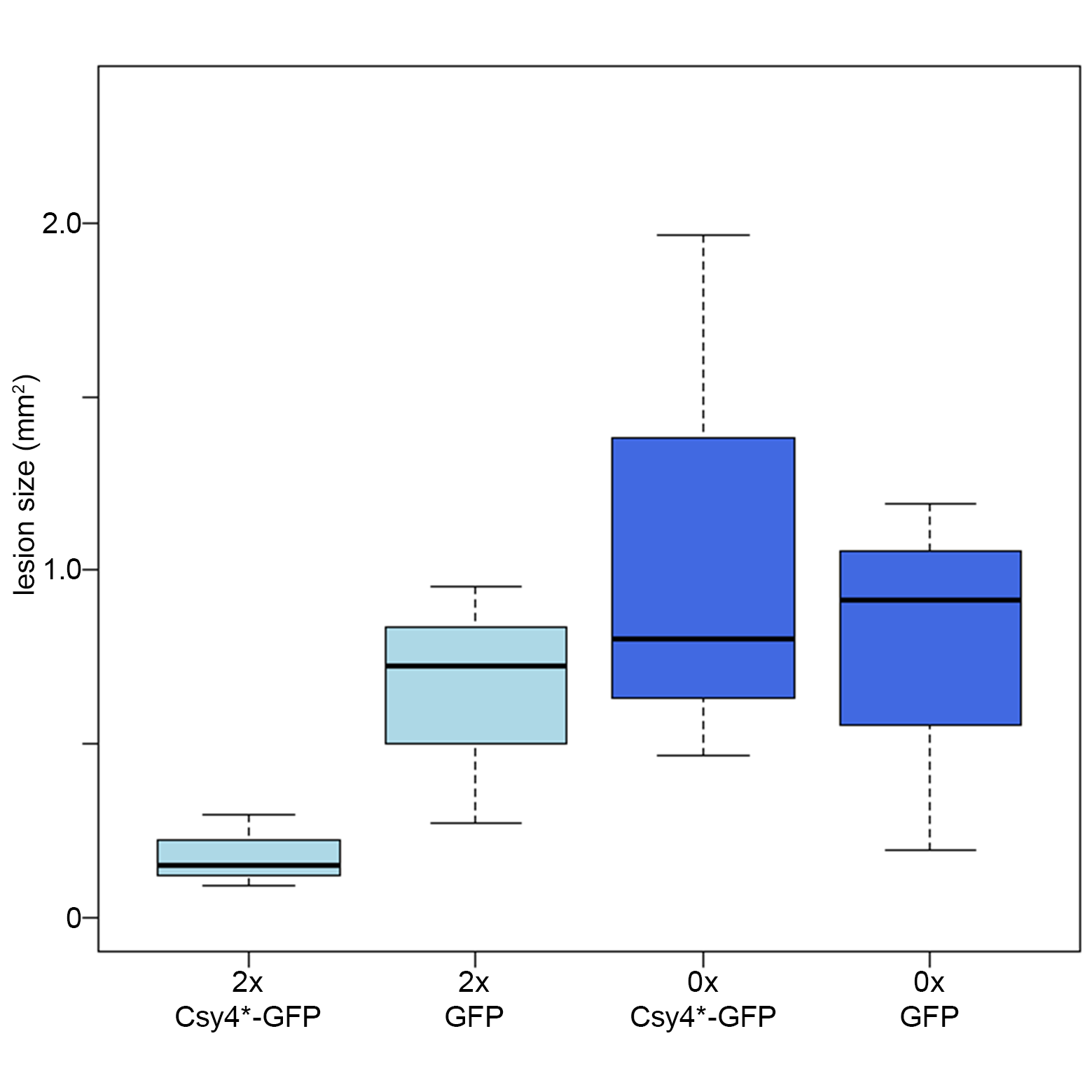

Supplement: S8 Fig — Lesion sizes of untagged (0x) or 2xcsy-tagged (2x) PVX.RFP-2A-CP at 6 days post inoculation. Virus was inoculated on leaves expressing either unfused GFP or Csy4*-GFP (2X/Csy4*-GFP: 85, 2x/GFP: 99, 0x/Csy4*-GFP: 93, and 0x/GFP: 100 infection sites; n = 3 independent experiments). Two-way ANOVA: The interaction between the two variables, number of tags versus expressed GFP construct, was significant (***; p = 1.13 × 10^-5^), however, pairwise Tukey test found no significant differences between treatments. (TIF) [file ppat.1013049.s008.tif]
