# Supplementary material for: Live-cell RNA imaging with the inactivated endonuclease Csy4 enables new insights into plant virus transport through plasmodesmata
Source: PLoS Pathog. 2025 Apr 9;21(4):e1013049. doi: 10.1371/journal.ppat.1013049 (PMC12052393; doi:10.1371/journal.ppat.1013049)
Supplement: Attachment — Submitted filename: Response to Reviewers.pdf [file ppat.1013049.s011.pdf]

Response to reviewers

We thank the reviewers for their careful analysis of our manuscript, which has helped us to address weaknesses and clarify our data presentation and conclusions. Responses to all reviewer comments are detailed below and we hope that these address all concerns raised.

Please note that all references to line numbers in the revised manuscript correspond to the version with tracked changes.

Reviewer's Responses to Questions

Part I - Summary

Please use this section to discuss strengths/weaknesses of study, novelty/significance, general execution and scholarship.

Reviewer #1: The manuscript by Burnett et al. described a new method for labeling viral genomic RNA based on the CRISPR-associated endonuclease Csy4. Although several other similar systems have been reported, this Csy4-based labeling system seems to be more sensitive possible due to its high affinity for its cognate stem-loop. Apart from this RNA labeling system, the results reported in the manuscript have not, in my opinion, yielded new insights concerning the cell-to-cell movement of plant RNA viruses.

We thank reviewer #1 for their careful consideration of our work. As we detail below, we do consider experimental evidence for the presence/accessibility of secondary structures in PD-localised viral RNA, the study of RNA localisation during movement complementation, and preferential RNA access of a cis-expressed RNA binding protein to provide novel insights. The reviewer has given more detailed reasons for their assessment below, which is where we also respond in detail.

Reviewer #2: This article presents an interesting piece of work in which the authors address a question that has remained elusive for a long time in plant virology: what is the physical entity that is transported through plasmodesmata? To do so, the authors use the high affinity of an inactivated bacterial endonuclease for its structured RNA motif.

We thank reviewer #2 for their positive assessment of our research. We would like to point out that our study also deals with RNA localisation during movement complementation, recruitment of replication complexes and presents findings with 'self-tracking' viruses expressing the RNA

reporter in cis.

Reviewer #3: The manuscript "Live-cell RNA imaging with the inactivated endonuclease Csy4 enables new insights into plant virus transport through plasmodesmata" by David Burnett and Mohamed Hussein et al. uses the inactivated endonuclease Csy4 to visualize viral RNA in living plant cells.

Unlike other live cell RNA imaging systems published before, this manuscript for the first time showns vRNA labeling inside plasmodesmata (PD) during virus infection. Based on this observation a range of conclusions are drawn and discussed. Among the conclusions is that folded RNA regions may still be present during transport through PD, PVX RNA may not be fully encapsidated during transport and replication proteins may be co-transported within the vRNP through the PD pore. Movement complementation experiments using the tobacco mosaic virus (TMV) movement protein (MP) are employed to demonstrate that also in the presence of the TMV MP PVX replication complexes are found close to PD. Finally the authors engineered a self-tracking virus expressing Csy4* and the cognate stem loops. While Csy4*-GFP expressing virus moved between cells, movement of the virus containing the stem loops was impaired.

The main novelties of this study are the application of the Csy4* system to label RNA in plant cells and the labeling of RNA inside the PD pore during virus infection. Consequently, the discussion of the potential implications of this finding for the mechanism of viral RNA movement and the nature and structure of the moving RNA-protein complex is a key point of the manuscript. Given the predominantly descriptive data obtained by confocal microscopy, the conclusions are naturally inferred from the observations. Thus, while it is certainly important to include possible conclusions drawn from the observations presented here on vRNA movement through PD and virus replication into current models, care must be taken not to over-interpret the observations.

We thank reviewer #3 for their careful analysis of our results. We have made a number of modifications to avoid overinterpretation of our data. As for reviewer 1, we respond to the detailed suggestions by reviewer 3 below. (We would like to clarify here that co-transport of replication proteins is not a conclusion of our study, we just point out that our data are compatible with previous publications suggesting this.)

Part II – Major Issues: Key Experiments Required for Acceptance

Please use this section to detail the key new experiments or modifications of existing experiments that should be absolutely required to validate study conclusions.

Generally, there should be no more than 3 such required experiments or major modifications for a "Major Revision" recommendation. If more than 3 experiments are necessary to validate the study conclusions, then you are encouraged to recommend "Reject".

Reviewer #1: (1) The idea that PVX RNA is not fully encapsidated during movement is possible but not new, also drawing such a conclusion based on the observation that PVX RNA granules were observed in the plasmodesmata is not sufficient. It is possible that Csy4-GFP binds to PVX RNA before it enters the PD, and its high binding affinity prevents its release from the RNA in the plasmodesmata channel. A direct comparison of the cell-to-cell movement efficiency between the combinations PVX.4xcys.RFP-2A-CP and PVX.4xcys.RFP-2A-CP + Csy4*-GFP may be helpful.

Whilst incomplete encapsidation of PVX RNA during movement has been proposed previously (Karpova et al., 2006 J Gen Virol; Tilsner et al., 2013 J Cell Biol), to our knowledge the data using the Csy4 system presented in this manuscript provide the first direct, in planta evidence. This is because for Csy4-GFP to bind to PVX RNA, secondary structures need to be present in the PD-localized RNA, which are incompatible with full encapsidation: The RNA affinity of Csy4 is mainly due to non-base-specific interactions of several Arg residues with the phosphate groups in the major groove of the RNA stem (Sternberg et al., 2012 RNA). Therefore, Csy4*-GFP recruitment into PD demonstrates the presence of secondary structures, which would be eliminated by complete encapsidation (Grinzato et al., 2020 Nat Chem Biol).*

The reviewer comments that Csy4-GFP could bind to the RNA before entering PD and then be unable to dissociate within the channel, and asks for a comparison of movement efficiency between a tagged virus with and without expression of Csy4*-GFP fusion protein. Whether Csy4 binds vRNA before it enters PD or after would not really make a difference in terms of the requirement for folded stem-loops. However, we have interpreted this as an argument that in a native PVX infection the RNA may actually be fully encapsidated with no secondary structures present - and that we are therefore only observing RNA with secondary structures inside PD due to the artificial situation of having introduced stem-loop tags and their subsequent stabilisation by binding of the high-affinity Csy4.*

We do include a comparison of a tagged virus with and without co-expressed Csy4-GFP as suggested by the reviewer (S8 (previously S4) Fig). This does show that Csy4 binding slows down the spread of a tagged virus (though not statistically significant for 2xcsy tags), perhaps by*

*inducing steric hindrance during transport. However, we also observed a statistically significant difference in spread between a 2x- and a 12x-tagged virus in the **absence** of Csy4 (Fig 5), which shows that the stem-loops **themselves** have a negative effect independent of Csy4 binding, which might not be the case if they were normally unfolded during movement.*

However, we do agree with the reviewer that from our data with an artificially tagged virus we cannot exclude full encapsidation occurring during movement of native PVX. Nevertheless, we <u>can</u> at least conclude that RNA secondary structures and associated incomplete encapsidation do not prevent the viral RNA from entering PD. We have therefore revised and more carefully worded our discussion in this regard (lines 668-675 in revised manuscript).

(2) The claim that the recruitment of VRC to PD is not virus-specific is also not well supported: Considering the cell-to-cell movement of PVX.ΔTGB.30k.4xcsy.RFP-2A-CP is slower than wild-type PVX (line 366), and I guess the percentage of PD that is associated with VRC in the cell infected by PVX.ΔTGB.30k.4xcsy.RFP-2A-CP should be lower than that in the cells infected by PVX.4xcsy.RFP-2A-CP. However, there is no such data in the paper. Fig.6F could be due to VRCs moving with the cell stream are hung up by the TMV MP multimers protruding at the PD, which allow the transport of viral RNA at a low efficiency. This is actually consistent with the previous results that the interaction between TGB2 and RdRp is required for efficiently recruiting VRC to PD (see doi: 10.1128/JVI.01635-18).

We interpret the reviewer's argument here to mean that slower movement of the complemented PVX.ΔTGB.30k virus could be due to reduced/abolished association of VRCs with PD and that where VRC association with PD is observed (Fig 6F) this might be due solely to chance encounters, rather than recruitment. This would mean that in actual fact there is no non-specific recruitment of the PVX VRC during movement complementation and we are over-interpreting the data.

However, VRC PD association was in fact widespread for PVX.ΔTGB.30k, i.e. Fig 6F is no rare outlier. The panel in Fig 6F is cropped from a larger image showing several more such PD-associated VRCs, now added as new S4 Fig C,D. This in turn was one of many such images we obtained.
Similarly, as can be seen in Figure 6E, there was Csy4-labelled viral RNA in 'cap' structures at most PD in the cell on the left just becoming infected (see also new S4 Fig A,B for infection in that cell). Thus, PD-associated VRCs were not a rarity during PVX movement complementation and there is therefore no indication from our data that reduced movement was caused by a failure to localise VRCs to PD.*

The reviewer further points out that in a native PVX infection, recruitment of VRCs to PD depends on interaction between TGB2 movement protein and replicase (Wu et al., 2019 J Virol), perhaps to suggest that in the absence of TGB2, as in our movement-complemented PVX.TGB.30k construct, VRC recruitment should not be possible. However, our suggestion that TMV 30k might mediate VRC recruitment indirectly by interacting with viral RNA is perhaps less contentious when considering that 1) TGB2 likely also interacts non-specifically with viral RNA like 30k (Hsu et al., 2009 Virol J; Wu et al., 2019 J Virol) and 2) the PVX replicase cannot be recruited to PD by TGB proteins alone, but only in the presence of replicating virus, i.e. viral RNA (Tilsner et al., 2013 J Cell Biol; Wu et al., 2019 J Virol). Therefore, some role of viral RNA in VRC recruitment seems likely even in a native PVX infection, and it is not too surprising if 30k - a PD-targeting and RNA binding protein that can recruit a nuclear-expressed mRNA to PD (Sambade et al., 2008 Traffic) - is able to do the same. We have revised the discussion to include these points (lines 375-377 and 694-697 in revised manuscript).

The reviewer also suggests that VRCs might be moving with cytoplasmic streaming and become trapped at PD due to the action of TMV 30k already located there. This touches upon the question of whether 30k arrives at PD already with a bound VRC 'in tow', or first reaches PD and then traps free, mobile VRCs there. In our view, these scenarios might not be mutually exclusive, and would not fundamentally differ in the mechanism of VRC recruitment (Tilsner and Oparka, 2012 Curr Opin Virol). However, as we had highlighted possible close spatial links between viral gene expression, replication and VRC targeting in our discussion, we have now modified this section to ensure a more balanced presentation of both possibilities (lines 704-706 in revised manuscript).

Alternatively, it is possible that TMV MP could interact with PVX RdRp or CP via a common yet unknown mechanism to recruit PVX VRC. The authors need to exclude all these possibilities.

When the reviewer suggests TMV 30k could interact with PVX RdRP or CP through a "common yet unknown" mechanism, in our view the hypotheses we present, of interaction via the RNA or host factors, actually fit that description. However, perhaps the reviewer was implying a direct protein-protein interaction between these proteins from unrelated viruses. To test this possibility, we have co-expressed 30k with either the PVX replicase or CP. In neither case are the PVX proteins recruited to PD by 30k (new S5 Fig). This is analogous to the cognate PVX TGB movement proteins, which cannot recruit CP or RdRP in the absence of infection (Tilsner et al., 2013 J Cell Biol). It is also in agreement with the absence of CP enrichment in PD in the movement-complemented PVX construct (Fig 6D). Therefore, direct interactions between TMV

30k and PVX RdRP or CP are less likely to be responsible for VRC recruitment. We have modified the results and discussion sections to reflect the additional data in Suppl S5 Fig (lines 433-438 and 694 of revised manuscript).

Reviewer #2: The work is well done and the article well written, although my main objection is that the conclusions that the authors draw from the results are not sufficiently supported by them. Even recognizing that the work is done with sufficient detail and meticulousness, there are three clear objections to it that make it require important modifications. i) the authors describe as new the highly sensitive RNA live-cell reporter based on an enzymatically inactive form of the small bacterial endonuclease Csy4, which binds to its cognate stem-loop with nanomolar affinity, but in fact they had already described it previously in a methodological article with a great level of detail (Burnett et al., 2020). In fact, in that article they already state that the inclusion of two tags is sufficient for a good image and here they use in several experiments constructions with 4 tags.;

We had been transparent about the previous Methods in Molecular Biology chapter in our cover letter to the editors when submitting to PLoS Pathogens and would like to point out that this chapter, which is referenced in the manuscript, does not show ***any*** *results and in particular does not mention the ability to detect RNA inside PD (which are never mentioned in the protocol). It deals mainly with cloning procedures (that are by now outdated due to the cheap price of gene synthesis) and general imaging settings. Half of the methods chapter is concerned with implementing the imaging system in a filamentous fungus rather than plants. Therefore, we do not agree that the previous methods chapter invalidates the novelty of the findings shown here. However, we have eliminated wording that describes the Csy4 system as "new", and "introduce" with "characterise" throughout.*

ii) I think the statement in the results section 'Csy4* labels viral RNA inside plasmodesmata' is too excessive and lacks conclusive evidence. To claim that when the signal of Csy4*-GFP concentrated in the inter-cellular cell wall space is clearly seen means that it is inside plasmodesmata seems to me to be weak. To claim that it is 'inside' and not 'associated' would require resolution at the level of electron microscopy;

We disagree with this assessment by the reviewer. Firstly, Csy4-GFP signal is not only seen in the intercellular wall space, but it co-localises there with TGB1 (S3 (now S7) Fig D), TGB2 (S3 (now S7) Fig E) and CP (Fig 3, S3 (now S7) Fig G), all of which have previously been shown to localise to PD during infection, including at TEM level for CP and TGB2 (Oparka et al., 1996 Plant*

J; Santa Cruz et al., 1998 Plant Cell, Samuels et al., 2007 Virology; Tilsner et al., 2013 J Cell Biol). By contrast, TGB3, which resides in membranous 'caps' adjacent to PD (Tilsner et al., 2013 J Cell Biol) is also localised adjacent to Csy4-GFP (S3 (now S7) Fig F).*

Secondly, determining PD localisation from co-localisation at confocal level is common (e.g. Thomas et al., 2008 PLoS Biol; Ishikawa et al., 2017 PLoS Pathog; Qin et al., 2024 PLoS Pathog), cell walls of mature epidermal pavement cells in Nicotiana benthamiana have a thickness of ~250-350 nm (e.g. see figures 2G and 5F in Tilsner et al., 2013 J Cell Biol) which is within the resolution limit of confocal microscopes, and localisations within and adjacent to PD can be clearly distinguished (e.g. Wei et al., 2010 PLoS Pathog; Tilsner et al., 2013 J Cell Biol; Levy et al., 2015 Curr Biol; see Figure panels 6E,F and S3F (now S7F) in this manuscript).

iii) It is difficult to assimilate and interpret that the construction untagged PVX.RFP-2A-CP rendered signicant PD labeling when compared with the tagged one; Indeed authors stated that the strong recruitment of Csy4*-GFP by PVX RNA even without csy tags facilitated co-localizations with transiently expressed PVX proteins. This suggests to me that the effect is being unspecific.

Firstly, to clarify, strong recruitment of Csy4-GFP by an untagged PVX construct occurred only when the Csy4 fusion was expressed from the virus* ***in cis****, and that is how all the co-localisations shown in S3 Fig (now S7 Fig) were obtained. When untagged virus was imaged with Csy4*-GFP expressed* ***in trans****, PD labelling was quite rare (whereas with a tagged virus it is nearly ubiquitous) and even then, the GFP signal in PD appeared weaker compared to cytoplasmic background than with a tagged virus (Fig. 3). We have now clarified this in the results section describing Fig. 3 (lines 266-268 in revised manuscript).*

We agree with the reviewer that when labelling occurs with an untagged virus, this is non-specific. As the strength of the Csy4-RNA interaction is mainly due to interactions with the RNA backbone in the major groove of the hairpin stem (Sternberg et al., 2012 RNA), we assume non-specific interactions to occur preferentially with RNAs containing secondary structures, the same way we also interpret the nucleolar enrichment of Csy4 (Fig. 1, new S1 Fig) and also of λN22 (Fig. 4G). In agreement with this, both nucleolar enrichment and PD localisation disappear when a Csy4 variant with mutations in the major groove-binding arginine residues (Csy4[mut]-GFP) is used (Fig 1F (now Fig 1C) and new S3 Fig).*

Localisations observed during imaging in the complex environment of living cells can be expected to reflect the steady state resulting from binding and dissociation events. Therefore,

even when the binding to non-cognate RNA secondary structures is orders of magnitude weaker than with the cognate stem-loop, it is likely to lead to a preferential enrichment compared with cell regions free of RNA, or containing less structured RNA. We have previously observed this also with a Pumilio-based RNA reporter (Tilsner et al., 2009 Plant J). We have now discussed the non-specific binding in more detail (lines 617-636 in revised manuscript), also in response to comments from reviewer 3 (see page 11 below).

Finally, authors speculate that the ability of Csy4* to detect TMV vRNA and PVX.ΔTGB.30k vRNA inside PD indicates that secondary structures remain present in vRNA during 30k-mediated virus transport. Based on complexes of 30k and vRNA formed in vitro it has been suggested that cooperative binding of 30k protein unfolds vRNA to enable passage through PD, and the 30k-vRNA complex is inaccessible to ribosomes. To demonstrate that Csy4* is accessible to the structured RNA an easy experiment would be to carry out EMSA assays of the corresponding construct and Csy4* in the presence and absence of TMV MP.

Our conclusions about the presence of RNA secondary structure inside PD during virus transport are based on Csy4 RNA affinity being predominantly due to interaction with the dsRNA stem of its cognate target (Sternberg et al., 2012 RNA; and see our response to reviewer 1 on page 3 above). There is no evidence that Csy4 binds ssRNA. Additionally, we have now also included data showing a Csy4 variant with mutations in the RNA stem-interacting Arg residues does not produce PD labelling of a tagged PVX (new S3 Fig).

Regarding presence of stem-loops in viral RNA despite unfolding by the cooperatively binding 30k protein, the original publication proposing this linearisation of RNA (Citovsky et al., 1990 Cell) actually predicted that secondary structures would remain, as 30k binds only single-stranded nucleic acids (see Figure 9 in Citovsky et al., 1990). When 30k-DNA complexes were subsequently visualised by TEM (Citovsky et al., 1992 Plant Cell), no unwinding of dsDNA regions was observed. Thus, our data are fully in agreement with these previous results.

As ribosomes scan ssRNA from the 5'end, the nontranslatability of 30k-bound RNA (Karpova et al., 1997 Virology) does not preclude the existence of unbound secondary structures further downstream in the genome. In fact, the authors noted that inhibition of translation occurred at relatively low 30k/RNA ratios of 100-150:1, at which presumably the TMV genome was not fully covered by 30k. Furthermore, our data are also consistent with experiments by the Heinlein group, who used stem-loop tagged, nuclear-transcribed mRNAs encoding 30k to show that the protein recruits its own mRNA to PD (Sambade et al., 2008 Traffic; Peña et al., 2021 Plant J). These papers did not draw explicit conclusions about the presence of secondary structures in

the RNA within plasmodesmata, but in all cases PD-localisation of stem-loop binding protein fusions was observed, i.e. stem-loops must have been present in PD.

Lastly, we also anticipate that the suggested EMSA experiments would not be straightforward. The cooperative binding of 30k would lead to a significant shift in itself and any further, smaller shift caused by binding of a limited number of Csy4 molecules would then still need to be resolvable.

Finally, one of the main conclusions is that the ability of Csy4* to label vRNA inside PD may be due to its higher affinity for its cognate target sequence compared with the other imaging systems. However, it is said that tight binding of Csy4*-GFP interfered with access to vRNA during replication or recruitment for movement. Both affirmations are difficult to reconcile.

The wording "tight binding of Csy4-GFP interfered with access to vRNA during replication or recruitment for movement" (lines 623-624 in original submission) had been intended to leave it open how and at what step the spread of the tagged, 'self-tracking' PVX constructs is impaired as we do not yet fully understand this (an area we are now actively focussing on). However, this seems to have prompted the reviewer to read our conclusions as contradictory or mutually exclusive. I.e., if Csy4 RNA binding is so strong that it can interfere with viral spread, then how is PD labelling possible, or why is movement not blocked when PD labelling is observed? We do not think that this is necessarily such a binary outcome in reality.*

Impaired spread could be caused by Csy4-binding directly interfering with RNA passage through PD. But this is not necessarily a given, as for instance, a BglG reporter was co-transported through PD with an mRNA encoding for TMV 30k protein tagged with 18 cognate stem-loops (Peña et al., 2021 Plant J), presumably a much stronger sterical hindrance than our 2xcsy tags (but also a different movement system and lower-affinity RNA binding protein). However, even if steric hindrance is the main cause, not all RNA passing through PD will necessarily be labelled and the degree of movement impairment could be related to the fraction of viral RNA that is bound by Csy4. Because PVX RNA probably continues to accumulate in PD even after infection of a neighbouring cell has been accomplished, strong PD labelling by Csy4-GFP could result either from a small fraction of bound RNA slowly accumulating, or from a majority of the RNA being bound, and may not necessarily be directly proportional to movement efficiency. We have now included a detailed consideration of these possibilities in our discussion (lines 724-743 in revised manuscript) though it does increase its length and complexity.*

Reviewer #3: In my opinion, the main concern about the data is that it is not clearly shown that indeed genomic RNA is seen in PD, or if rather the subgenomic RNAs expressed during virus infection are bound by the Csy4*-protein when expressed in trans or from the virus. The conclusions drawn on virus movement are based on the assumption that genomic viral RNA is labelled. However, the stem loops and the Csy4*-GFP coding sequence are located in a region, where a subgenomic RNA is made (see ref 109).
Have the authors tried to experimentally determine whether genomic or subgenomic RNAs are preferentially bound? I imagine that an experimental approach to demonstrate which RNAs are bound by the protein is very difficult, as it would require e.g. immunoprecipitations of minute amounts of the Csy4*-GFP-expressing virus from infection sites and the determination of the nature of the bound RNAs. However, these experiments could also give an answer to the question, which proteins are in the complex. By contrast, as the tagged virus PVX.2csy.RFP-2A-CP moves systemically, agroinfiltrated Csy4*-GFP could be used in systemic tissue to immunoprecipitate the CSY4*-GFP-RNA complex and determine, whether genomic or subgenomic RNAs are preferentially bound.

The reviewer raises an interesting point here, which we have now included in our discussion (lines 609-616 of revised version). We would assume that Csy4-GFP generally binds all viral RNAs containing the stem-loops, both genomic and subgenomic. However, in PVX, where the CP is required for movement and co-localises in the PD, the encapsidation signal is located near the 5' end of the genome (Kwon et al., 2005 Virology; Verchot-Lubicz et al., 2007 J Gen Virol; Petrova et al., 2015 Biochimie) and therefore absent from subgenomic RNAs. It is has also been shown that this genomic region is required for movement (Lough et al., 2006 Virology). To our knowledge, there is so far no evidence for encapsidation of PVX subgenomic RNAs. Therefore, we assume that when Csy4*-GFP is localised to PD, this is in association with genomic PVX vRNA.*

As the reviewer assumes, it would be hard to definitively prove this experimentally, as not only would it require identifying bound RNAs after immuno-precipitation, but also distinguishing those bound by cytoplasmic Csy4-GFP versus PD-localised Csy4*-GFP. Sampling systemic leaves would not change this in our view, as the virus will initiate fresh infections in systemic tissue and therefore also produce subgenomic RNAs. We have also tried in a different project to insert small additional sequences into the PVX 5'UTR and found it rendered the virus non-infectious (unpublished data, in agreement with Kim & Hemenway, 1999 RNA). Therefore, we did not attempt to move the csy stem-loops upstream of the replicase ORF, where they would be present only in the genomic RNA. Inserting stem-loops immediately downstream of the*

replicase may also result in disruption of the TGB1 subgenomic promoter (Kim & Hemenway, 1997 Virology; Kim & Hemenway, 1999 RNA).

However, in the case of TMV and the TMV 30k movement-complemented PVX, where no encapsidation is required for movement and 30k has already been shown to recruit its own mRNA (Sambade et al., 2008 Traffic) we can indeed not be certain if signal in PD is due only to genomic vRNA or may also include subgenomic RNAs. Nonetheless, if subgenomic RNAs are present and labelled within PD, the 30k-mediated transport mechanism would probably not be any different than for genomic RNA, so any conclusions about presence of stem-loops for instance would be unaffected.

The data in Figure 1D indicate that Csy4*-GFP also binds to cellular RNA in the absence of the cognate stem loops (RNA-rich nucleolus and granules). In Fig. 2D, 3E, Fig. 7 A-D it is shown that Csy4*-GFP efficiently binds to vRNA in the absence of stem loops. However these findings are not sufficiently discussed. I would have liked to see a discussion on the specificity of binding to RNA other than the target RNA or the occurrence of the specific RNA sequences resembling the cognate stem loops in viral and plant cellular RNA.

To start with a clarification: Csy4-GFP localisations in Fig 2D and 3E do not show "efficient" binding to untagged vRNA. Fig 2D shows a mostly diffuse signal inside the PVX VRC, which is a cytoplasmic structure also labelled by free GFP (Ju et al., 2005 Plant Physiol), and only a very faint recruitment to the typical 'whorls' (Tilsner et al., 2009 Plant J), which can be seen much more strongly labelled with tagged viruses in Fig 2A-C. Similarly, Fig 3E shows only a weak PD enrichment and PD labelling was very rare with an untagged virus, whereas it was nearly ubiquitous for tagged PVX constructs. We have now made this clearer in the descriptions of these results in the main text (lines 282-284 in revised version). Thus, Csy4*-GFP bound to untagged viral RNA efficiently* ***only*** *when expressed in cis from the viral genome (as in Fig 7B-D (now Fig 7C-E) and S3 (now S7) Fig.*

However, we now discuss more extensively how non-specific binding to non-cognate RNA secondary structures may still lead to preferential enrichment within the cell despite orders of magnitude lower affinity (lines 617-636 in revised version). This also includes discussion of possible reasons for why non-specific RNA binding does not also result in PD localisation of Csy4-GFP in uninfected tissue, which the reviewer had raised in their comment after next.*

The data in Fig 1 F Csy4*[mut]-GFP are interesting, as this indicates absence of localization to RNA-containing subcellular structures for this mutant. Why was this mutant not employed as

control for experiments with viral RNA? It would be nice to see the absence of signal in PD when this mutant is co-expressed with virus containing stem loops, complementary to the data shown in Fig. 3 C and D.

We thank the reviewer for this suggestion and have now included these data as new S3 Fig. As expected, the mutated Csy4 does not localise to PD even when the virus is tagged. The corresponding results descriptions have been added to the manuscript in lines 255-257 and 269-270 in the revised version.

Moreover, how do the authors interpret the results in Fig. 3 E,F? In line 254 – 256 the authors say that no Csy4*-GFP signal in PD was seen in the absence of virus infection. So I suppose the authors conclude that the PD labeling of Csy4*-GFP during PVX infection without stem loops results from RNA labeling of viral RNA inside PD. Why would endogenous RNA moving through PD not be labeled? Do the authors think this is because of high concentration of virus RNA inside the PD or maybe because viruses dilate PD and only dilated PD can accommodate the Csy4*-GFP inside the PD pore?

As the reviewer suggests, we suspect that the lack of Csy4-labelling of PD in the absence of infection may have to do with the relative abundance of endogenous mobile RNAs compared with vRNA. In non-vascular tissue, there may be only a limited number of RNA species being transported, involving specific targeting mechanisms (Luo et al., 2024 Nat Plants) and these may be significantly less abundant than viral RNAs, as well as being transported with lower efficiency than provided by viral movement proteins. This has now also been included in the discussion (lines 631-636 in revised manuscript).

Regarding the role of viral gating, some fluorescent fusion proteins of a similar size to Csy4-GFP or larger can move through PD non-specifically (Oparka et al., 1999 Cell). However, they do not accumulate inside the channels in the process. Therefore, we assume that localisation of Csy4*-GFP in a punctate pattern at PD depends on the presence of a trafficked RNA that it can bind to.*

Line 394: “vRNA was also observed inside PD but with no co-localisation of RFP-CP (Fig 6D, E), unlike native PVX but as expected for movement mediated by TMV 30k” The authors use this difference to the PVX-based observation to demonstrate that the Csy4* system is able to confirm the postulated and previously reported movement mechanisms for PVX (with CP) vs. TMV (without CP). However, the authors do not discuss the faint RFP-2A-CP signal inside the PD

in the Figure 6D. Moreover, in Figure 6E, showing the cell at the leading edge of infection I am not convinced that the cell, into which the virus moves, expressed the RFP-2A-CP protein.

Regarding the faint RFP signal within the cell wall space in Fig 6D, this is of lower intensity than the neighbouring cytoplasm, whereas in native PVX infection, CP is clearly enriched in PD (e.g. Fig 3, S3 (now S7) Fig G). We interpret this as small amounts of fluorescent RFP-2A-CP, or free RFP-2A (resulting from 2A-mediated separation), entering the virus-gated PD, perhaps still binding the origin of assembly, but prevented from full encapsidation by the cooperatively binding 30k (Citovsky et al. 1990 Cell). We have now clarified this in the manuscript (lines 426-429 in revised version).

Regarding the image in Fig 6E, this shows a projection of just two z-sections focussing on the mid-level cell periphery. We have now added a maximum projection of the entire z-stack as a new S4 Fig A, B, which shows the beginning RFP expression in the cell in question.

Line 484 to 489: This paragraph and the Figure S4 is unclear to me. The difference in lesion size between tagged virus in Csy4*-GFP expressing tissue compared to the other conditions is described as insignificant, but the infection sites according to the box plot are approximately three fold smaller. Fig. S4 legend says that ANOVA found a significant difference. Agroinfiltration appears to affect lesion size, as lesion size of the two-times tagged construct in Figure 5C was approximately two-fold the size of lesions of untagged or tagged virus in the presence of GFP. Could it be that the binding of the vRNA by the Csy4*-GFP transiently expressed in cells ahead of the infection front hinders spread of infection or replication for the condition, where the tagged virus is analyzed?

*To clarify, the ANOVA performed on the data presented in S4 (now S8) Fig showed a **significant interaction** between the two variables (number of tags and infiltrated GFP construct), but **no significant differences** in any pairwise comparisons. However, we noticed an error in the p-value for the interaction in the figure legend, which has now been corrected (line 73 in revised Supplementary Materials file). We have modified the description of these results in the main text to make this clearer there as well (lines 538-541 in revised manuscript).*

That being said, we have been very conservative in our statistical analysis. To avoid biologically insufficiently independent pseudo-repeats, the ANOVA and post-hoc test were done with n = 3 (experiments), not lesion numbers. We agree that there is a clear trend and likely if we had compared viruses with more stem-loop tags, would have found a statistically significant difference. So, we agree that transiently expressed Csy4-GFP slightly hinders the spread of the*

infection, though this effect is mild – after all, RNA imaging is based on overlap between the infection site and the agroinfiltrated are. However, the inhibition becomes severe when a 'self-tracking' virus is tagged. In this context, we had already mentioned the inhibitory effect observed by the reviewer (lines 615-616 in original submission, now lines 717-718 in revised version). We have now expanded the discussion of the possible mechanism(s) by which Csy4 binding might interfere with the spread of tagged viruses (lines 724-743 in revised manuscript).

In as far as lesion sizes in Fig 5C and S4 (now S8) Fig can be compared directly (the experiments were conducted ~1 month apart), we agree that a negative effect of agroinfiltration itself seems plausible. However, that is why in S4 (now S8) Fig, expression of free GFP by agroinfiltration was chosen as a negative control to normalise for any such effect.

Fig. 4 and discussion about the sensitivity of the different RNA labeling systems lines 547 to 553. Indeed, as is discussed, it seems likely that the other systems should be able to detect RNA inside PD during virus infection and that the lack of PD labeling by the other systems may be related to the sensitivity, which could be increased when more stem loops are used. However, I would like to mention that the cells shown in B display much less cytoplasmic labeling of the RFP protein compared to the cells in D,F and H. The data as shown might therefore suggest that B focusses at a cell more at the leading edge of infection, where RNA movement occurs, compared to the other infection sites, which look as if both neighboring cells are already fully infected. Maybe the legend should state that cells at comparable positions inside lesions were analyzed.

We want to point out that we have never observed any PD labelling with the other imaging systems, irrespective of where we imaged within the infected tissue. However, as we were unsure retrospectively, where exactly within a lesion each image was taken, we have now repeated these observations and replaced the panels in Fig. 4B, D, F, H with new images. PD labelling with Csy4-GFP was most easily observed at the leading edge of the infection sites, as surmised by the reviewer. We have now ensured that the images representing the other systems are all from comparable locations at the lesion edge, which is also stated in the figure legend.*

Additional discussion would be needed to explain why there should be a higher sensitivity of the other systems to label RNA at PD upon ectopic expression of the RNA and proteins (references 54 and 55) vs. expression during infection.

The other systems were not more sensitive towards nuclear-transcribed mRNAs compared to viral RNAs, rather these prior publications used between 12 and 18 stem-loops, whereas we

used only 2 stem-loops (for the TMV constructs). This is described in lines 514-520 in original submission (now lines 569-575 in revised manuscript).

Part III – Minor Issues: Editorial and Data Presentation Modifications

Please use this section for editorial suggestions as well as relatively minor modifications of existing data that would enhance clarity.

Reviewer #1: (1) Fig. 4: The panels D, F, and H are not typical as there is no plasmodesmata or VRCs can be found.

These panels do not show VRCs because they are focused on the cell periphery. However, they are typical precisely because they do not show plasmodesmata, as we have never observed any PD labelling with the other imaging systems, only with Csy4. Note that in response to a reviewer #3 comment in Part II, we have replaced panels B, D, F and H with new figures all taken at comparable positions at the lesion edge.*

(2) Fig. 5 and cognate context: The size of the bands of PVX.12xcys.RFP-2A-CP in Fig. 5G is not equal (lanes 3, 9, and 14). Also, lane 10 contains two bands. Therefore, too much hairpin is genetically unstable, in my opinion. Thus, it needs very carefully consideration to draw the conclusion that multiple csy tags have only a moderate negative effect on viral infectivity.

Thank you for highlighting these details. What we had meant was that none of the 12x tagged viruses showed bands of the same size or lower than the 2x virus, suggesting that none of them lost stem-loops entirely. However, the reviewer is of course correct that the RT-PCR results suggest the stem-loops where not 100% stable either. We have now re-worded the description of these data more carefully to reflect this (lines 343-347 in revised version).

Reviewer #2: Line 27, abstract: it is said "..movement proteins which bind nucleic acids and target and dilate plasmodesmata". This is a generalization of the MPs since not all of them have nucleic acid binding properties (e.g MPs of GFLV, CPMV, CLRV, etc..)

We have deleted this statement from the abstract (line 30 in revision), and re-worded the corresponding section in the introduction that the reviewer also highlighted (see response to comment after next below).

Lines 30-32, abstract; "Here, we describe a new, highly sensitive RNA live-cell reporter based on

an enzymatically inactive form of the small bacterial endonuclease Csy4, which binds to its cognate stem-loop with nanomolar affinity". See general comments above.

We assume this refers to reviewer #2's comments about the previously published methods chapter, which we have addressed above (page 6). As stated there, we have now removed the word "new" throughout, and replaced "introduce" with "characterised".

Line 67: or multiple MPs, nevertheless, some common features have emerged: 1) nucleic acid binding.
See above. Not all MPs bind nucleic acids. Most MPs forming tubular structures have not the capability to bind n.a.

We have re-worded this section to make it clear this does not apply to all MPs, and also lead the list of common features with PD targeting, rather than nucleic acid binding (lines 70-73 in revised version).

Line 81, when stating that capsid proteins (CPs) often preferentially interact with secondary structures in their cognate viral (v)RNA it is surprising that the paradigmatic and best studied example of AMV (or ilarviruses)-3'UTR was not cited.

We now include a reference to the AMV work (Zuidema & Jaspars, 1985 Virology), as well as a more recent review of plant RNA virus packaging mechanisms (Rao et al., 2014 Curr Op Virol) (new references [17-18]).

Line 86, many MPs are also able to non-specifically transport unrelated viruses.
It seems that some of the works that better describe this scenario have been forgotten.

As we were not aiming for a comprehensive overview of plant virus movement complementation literature, we had cited here a review that summarises many of these papers (Latham and Wilson 2008 Mol Plant Pathol) and one extreme example of 30k complementing movement of a non-plant virus. A further review summarising more recent movement complementation results was just published (Morozov & Solovyev, 2024 Front Plant Sci), which we have now added (new reference [24]). The section specifically describing the choice of TMV 30k for complementation (lines 357-361 and 365-366 in original submission, now lines 384-387 and 391-392 in revised version) then cites several primary publications that are directly relevant as they describe PVX complementation by TMV 30k.

lines 160 and following; The description of Figure 1 is inappropriate. Figures 1B and 1D appear

before 1A and 1C in the text. Modify the figure or change the order of description in the text. The same applies to Figure 4

In case of Fig 1, we have now removed panels A-C to a separate supplementary figure (new S1 Fig) in response to a comment from reviewer #3 (see page 21 below). The description of these figures in the main text was adjusted accordingly, and now matches the order of the panels in S1 Fig and Fig1 (lines 165-173 in revised manuscript).

For Fig. 4, we have changed the order of the text to match the figure panel numbering (lines 299-308 in revised version).

Lettering of Figure S1 does not match what is described in the legend. Figures are referenced as A to I whereas legend is only referenced for rows (A, B, C).

Many thanks, we have now corrected this error in the figure legend.

Line 212 I wonder why authors use Potato virus X tagged with four csy stem-loops if they already showed that two csy stem-loops are sufficient for imaging as described in their methods paper (Burnett et al 2020)

Firstly, whilst the Burnett et al. 2020 methods chapter mentioned that two stem-loops are sufficient for imaging, it did not actually show any results demonstrating this. When we begun using the Csy4 system, we had generated a few different tagged PVX constructs, including 2xcsy and 4xcsy. We started by testing the 4xcsy-tagged virus, including when trialling the nuclear-targeted Csy4-NLS-GFP construct, which we soon stopped using. Subsequently, we did not observe any qualitative differences when imaging 2x and 4x tagged viruses with the Csy4*-GFP construct and used them interchangeably for a while. Thus, we selected images from 4xcsy-tagged virus to compare the nuclear and cytoplasmic reporters for "historic" reasons, but then show the corresponding VRC image with 2xcsy in panel C.*

Line 215. ... at medium to late infection stages... Please, be more specific in this context. Readers could not remember how dpi are you talking about.

X-bodies are typically visible ~1 cell layer behind the infection front, within ~1 day of infection and we have now re-worded the sentence to include this information.

Line 243. however even in the absence of cognate stem-loops in the viral genome, the Csy4*-

GFP reporter still weakly showed the characteristic localisation to 'whorls' (Fig 2D). This seems quite anomalous. See my general comments

In response to the reviewer's concerns about the non-specific labelling of untagged RNA in Part II above (page 7), we have clarified the descriptions of these results in the main text (lines 250-252 in revised manuscript) and added a more detailed discussion of the non-specific binding (lines 617-636 in revised version). These revisions also apply to the point here. Additionally, in response to reviewer #3, we now also include the RNA binding-deficient Csy4[mut]-GFP construct as a control (new S3 Fig), which does not label 'whorls' even when the virus is tagged.*

Line 248 I think the statement in the results section ' Csy4* labels viral RNA inside plasmodesmata' is too excessive and lacks conclusive evidence. To claim that when the signal of Csy4*-GFP concentrated in the inter-cellular cell wall space is clearly seen means that it is inside plasmodesmata seems to me to be weak. To claim that it is 'inside' and not 'associated' would require resolution at the level of electron microscopy.

The reviewer had raised this concern also in Part II, where we have offered a detailed response (page 6) to defend our assessment that the confocal resolution is sufficient to conclude that the signal enriched within the otherwise non-fluorescent cell wall space is from within plasmodesmata.

255 When untagged PVX.RFP-2A-CP was used for infection, only very faint PD labelling with Csy4*-GFP was detectable (Fig. 3E,F). I wouldn't day that. Again, same concern as previous comments.

We are unsure here if "same concern as previous comments" refers to the interpretation of cell wall-inserted fluorescent punctae as being localised within PD (see response in Part II), or the fact that untagged virus produced a weak Csy4-GFP signal in PD. Assuming the latter, with a tagged virus, labelled PD were numerous and very easily observed. By contrast, with an untagged virus, they were very rare and the GFP signal in PD less enriched compared with adjacent cytoplasm than for tagged viruses. We have modified the description in the results to be clearer (lines 266-268 in revised manuscript).*

Lines 267-8. One of 'only' must be removed

Thank you, this sentence has been re-worded in response to the reviewer's concerns about labelling of untagged virus, and the duplicated "only" was eliminated in the process (now lines 266-269 in revised manuscript).

Line 284, λN22

Thank you, corrected (now line 303).

Lines 291-2 Authors stated that only Csy4*-GFP could detect vRNA in plasmodesmata, indicating that it is the most sensitive amongst these RNA imaging systems, but I think it is also possible to observe PD with λN22.

The reviewer is correct that λN22 could detect a nuclear-transcribed TMV movement protein mRNA inside PD. However, this was with 16 boxB stem-loop tags (Peña et al., 2021 Plant J), described in our discussion (previously lines 517-520, now lines 572-575 in revised version). By contrast, our experiments compared viruses with only two stem-loops, as stated in lines 278 and 290 in original submission (now lines 293 and 308) directly before the lines in question. We had also already acknowledged that as PVX tolerated insertion of 12 stem-loops, imaging of vRNA inside PD with λN22 should also be possible when more than two stem-loops are used (lines 552-553, now lines 607-608 in revision).

383, 392, 165k-GFP replicase marker located in 'caps' at the entrances of plasmodesmata labelled with aniline blue-staining of callose.
How are the authors sure that this is the PD entry?

What we meant by "PD entrances" was that the fluorescent signal was adjacent to, but not overlapping with the callose PD marker. For PVX moving with its native TGB proteins, we have previously shown by EM and 3D-SIM super-resolution imaging that the peripheral membranous 'cap' structures are adjacent to PD entrances (Tilsner et al., 2013 J Cell Biol). As here, movement was complemented by TMV 30k and we did not conduct EM imaging, we have re-worded this sentence to just speak of "peripheral structures adjacent to PD" (now lines 429-430).

396-8. The recruitment of PVX replication complexes by TMV MP would seem to be expected given that Sambade et al had already demonstrated that MP:mRFP as well as its transcript accumulate in PD suggesting that MP has the ability to target its own transcript to the channel. Thus, the targeting of RNA molecules to PD rather represents a sequence-non-specific function of the MP protein because MP has sequence-non-specific nucleic acid-binding activity and is known to facilitate the intercellular spread of other RNA molecules, such as the genomes of unrelated RNA viruses and RNA molecules implicated in the spread of RNA silencing .

We agree entirely with the reviewer that the non-specific recruitment of PVX RNA by TMV 30k is expected based on the established properties of the 30k protein and its documented ability to complement PVX movement. This is both how we introduce the choice of 30k for these experiments and discuss our results. However, to our knowledge, this is the first time that RNA live cell imaging has been used to study movement complementation, and more importantly, from the ability of 30k to transport unrelated viruses one cannot draw any conclusion about whether the replicase of an unrelated virus would also be recruited to the vicinity of PD, which we have tested here.

488 whilst Csy4*-GFP binding to csy stem-loops seems to have a negative impact on lesion size, it is only the specific combination of viral Csy4*-GFP-T2A expression in cis from a tagged vRNA that severely disrupts viral spread. A 'self-tracking' PVX construct demonstrates preferential access to vRNA when Csy4*-GFP is expressed in cis
How can it be stated preferential access to vRNA when Csy4*-GFP is expressed in cis if the untagged vRNA has similar signal intensity?.

Our conclusion that cis-expression of Csy4-GFP seems to provide preferential access to the vRNA is based on two observations. 1) PD signal for untagged virus is much stronger for cis-expression than for trans-expression. 2) Impairment of spread of a 2xcsy-tagged virus is mild for trans-expression and severe for cis-expression.*

The reviewer's question here, comparing the untagged and tagged 'self-tracking' viruses, implies that signal should also be stronger for the tagged virus. However, this may not be so straightforward. We have argued in a previous response to this reviewer that PD signal of Csy4-GFP might not be directly proportional to movement efficiency because different fractions of RNA can be bound by Csy4 and accumulation in PD may be gradual (see page 9 above and lines 728-734 in revised discussion). In the VRC, co-compartmentalisation of Csy4*-GFP expression with viral RNA might lead to strong association with 'whorls' even if the binding is weaker for an untagged virus.*

Reviewer #3: Line 159: revise sentence. I guess it should be either "each" or "both"

Thank you, we have corrected this sentence (now lines 1631-64).

Line 161: Fig 4A should be mentioned in the text before Fig 4B or the order in the Figure changed.

Line 163: Fig 4A to C should be mentioned in the text before Fig 4D or the order in the Figure changed.

Based on the line numbers, in both instances above the reviewer is presumably referring to Fig 1, rather than Fig 4 panels. In response to the reviewer's next comment below, we have now removed panels A-C to a new S1 Fig. Additionally, we have revised the text so that the text order now matches the figure/panel order in S1 Fig and the revised Fig 1 (lines 165-173 in revised manuscript).

Please note that for Fig. 4, we have also changed the text so that the description of results matches the panel numbering (lines 299-308 in revised manuscript).

Fig. 1: The results of the N-terminally tagged Csy4* and of the nuclear targeted Csy4* shown in Fig 1 A,B, and C are not relevant to the conclusions taken and could be presented as supplementary data.

As requested, we have removed these panels to a new S1 Fig. Accordingly, references to the figures in the main text have been updated and the numbering of the other supplementary figures has changed.

Fig. 2: Why is there only such a weak magenta signal for the CP in C? I would expect a signal similar to the one shown in Fig. 2A in 2C. Do the two stem loops avoid localization of the CP in the X-body or is packaging inhibited by the two stem loops?

We did not observe any effect of stem-loop tags on CP localisation or accumulation, and if there were any interference from the stem-loops - which are inserted far downstream of the encapsidation signal (see first response to reviewer #3 on page 10 above) - it would be expected to be more pronounced for the 4xcsy-tagged virus in Fig 2A.

The variations in RFP signal intensity in Fig.2 are just random. The RFP-CP signal varies significantly across a lesion and gain settings were not always consistent, but adjusted for the brightest regions in an image and by different experimenters. Images were selected for use in the figure based on good contrast in the GFP channel to show the 'whorls', with no consideration of the RFP signal.

Line 284: the "Lambda" symbol is not shown properly.

Thank you, this has been fixed in the revision (now line 303).

Lesion size experiments: It is unclear how many lesions were analyzed in the different experiments

We have added the total lesion numbers to the relevant figure legends (lines 354-355 and 485-489 in revised manuscript, and 70-71 in revised Supplementary Materials). In the process, we also discovered some small calculation errors in the numbers of lesions/µg DNA for both tagged 'self-tracking' viruses, and in the percentage of single-celled infections sites for PVX.2xcsy.Csy4-GFP-T2A-CP. These have also been corrected in the text (lines 522-525 in revised manuscript).*

Line 420: Fig. 7 A-J should be mentioned before Fig. 7K or the Figure should be restructured.

We agree with the reviewer that it makes more sense to present the Western blot first and have re-structured the figure accordingly (Western blot panel K has become new first panel A, and microscopy images in panels A-J have been shifted and became panels B-K).

Line 433: the representative infection site in E shows only one cell. Maybe this should be mentioned in the legend.

This is because 68% of lesions of this tagged virus were single-celled. We have added "single-celled" to the description in the legend (now panel F), and the corresponding "small multi-cellular" for I (previously panel H) (lines 477-479 in revised manuscript).

Fig. 7 M and N: The quantified lesion sizes in M and N for the non-tagged virus are different in size, approximately 2 mm2 in (M) vs. 1 mm2 in (N). Why is that? Were the lesions analyzed in different leaf ages or at different times after infection? I did not find the corresponding information.

These experiments were conducted with identical parameters (leaf age, time after infection) but at different times of the year (winter for M and summer for N). This likely caused some variations in light intensities and temperature in the glasshouse, resulting in some variation in spread of the control virus, as the reviewer has noticed. This is why we presented these experiments as separate graphs and did only pair-wise statistical comparisons for each. However, the upstream-tagged virus showed predominantly (68%) single-celled infections sites at the same time as the untagged control virus had <u>larger</u> lesions. On the other hand, the downstream-tagged virus had small, but mostly (75%) multi-cellular lesions at the same time as the control virus had <u>smaller</u> lesions. Therefore, it is unlikely that the upstream-tagged virus showed single-

celled lesions only due to the influence of climate. We have now added this information to the Figure 7 legend (lines 490-496 of revised manuscript).
