# Supplementary material for: Live-cell RNA imaging with the inactivated endonuclease Csy4 enables new insights into plant virus transport through plasmodesmata
Source: PLoS Pathog. 2025 Apr 9;21(4):e1013049. doi: 10.1371/journal.ppat.1013049 (PMC12052393; doi:10.1371/journal.ppat.1013049)
Supplement: Attachment — Submitted filename: Response to Reviewers R2.pdf [file ppat.1013049.s012.pdf]

Response to reviewers

We thank the reviewers for their positive responses to our revisions and have implemented all further changes they have suggested. These are briefly detailed below.

Reviewers' Comments:

Reviewer's Responses to Questions

Part I - Summary
Please use this section to discuss strengths/weaknesses of study, novelty/significance, general execution and scholarship.

Reviewer #1: The manuscript has been improved significantly in my opinion. Therefore, I will suggest a minor revision this time.

We thank the reviewer for their positive response.

Reviewer #2: Already described in the original version

Part II – Major Issues: Key Experiments Required for Acceptance
Please use this section to detail the key new experiments or modifications of existing experiments that should be absolutely required to validate study conclusions.

Generally, there should be no more than 3 such required experiments or major modifications for a "Major Revision" recommendation. If more than 3 experiments are necessary to validate the study conclusions, then you are encouraged to recommend "Reject".

Reviewer #1: NO.

Reviewer #2: In this paper I raised three main objections that the authors should address before the publication of the manuscript. Most of these were related to the lack of consistency in some results that made the conclusions based on weak demonstrations, e.g. the RNA label inside the plasmodesma and the non-specificity of the observed label. Most of their answers, if not all, are well argued and they have added some new experiments that clarify the proposed scenario of intercellular transport. The issues related to the specificity of the process (TMV MP) are now better explained in the discussion. They have added the RNA binding-deficient Csy4*[mut]-GFP construct as a control (new S3 Fig), which does not label 'whorls' even when the virus is tagged.

We thank the reviewer for their positive evaluation of our revisions.

Part III – Minor Issues: Editorial and Data Presentation Modifications
Please use this section for editorial suggestions as well as relatively minor modifications of existing data that would enhance clarity.

Reviewer #1: 1. The virus names should not be italic and not capitalized for the first character, e.g., potato virus X but not Potato virus X.

We have now corrected the formatting of all virus names (lines 32, 35-37, 115, 128-129, 132, 425, 432, 738, and 771-772 in revised manuscript (tracked version)).

2. line 354, TVCV may not a good example here as I cannot find a figure or description about the localization of VRC to PD in this paper; instead, maybe these two papers (doi: 10.1128/jvi.01898-19; 10.1111/tra.12768)

The work on TVCV we had cited (reference 72) does describe PD-adjacent ER-derived structures as replication sites. However, upon re-reading, we agree with the reviewer that this appears to be based on similarities with TMV rather than any actual localisation of the TVCV replicase. As PVX and TMV are directly relevant to our results, whereas TVCV and turnip mosaic virus (the focus of the first of the two references suggested by the reviewer) are not, we have replaced the mention of TVCV with "and other plant viruses", and replaced reference 72 with the second paper suggested by the reviewer, a general overview of targeting of VRCs to PD.

Reviewer #2: All minor objections have been corrected and/or answered.

We thank the reviewer for their positive response to our revisions.
